# Ventral hippocampal afferents to the nucleus accumbens regulate susceptibility to depression

**DOI:** 10.1038/ncomms8062

**Published:** 2015-05-08

**Authors:** Rosemary C. Bagot, Eric M. Parise, Catherine J. Peña, Hong-Xing Zhang, Ian Maze, Dipesh Chaudhury, Brianna Persaud, Roger Cachope, Carlos A. Bolaños-Guzmán, Joseph Cheer, Karl Deisseroth, Ming-Hu Han, Eric J. Nestler

**Affiliations:** 1 Fishberg Department of Neuroscience, Icahn School of Medicine at Mount Sinai, New York, New York 10029, USA; 2 Department of Psychology and Program in Neuroscience, Florida State University, Tallahassee, Florida 32306, USA; 3 Department of Pharmacology and Systems Therapeutics, Icahn School of Medicine at Mount Sinai, New York, New York 10029, USA; 4 Department of Anatomy and Neurobiology, University of Maryland School of Medicine, Baltimore, Maryland, 21201, USA; 5 Departments of Bioengineering and Psychiatry and Behavioral Sciences, Stanford University, Stanford, California 94305, USA

## Abstract

Enhanced glutamatergic transmission in the nucleus accumbens (NAc), a region critical for reward and motivation, has been implicated in the pathophysiology of depression; however, the afferent source of this increased glutamate tone is not known. The NAc receives glutamatergic inputs from the medial prefrontal cortex (mPFC), ventral hippocampus (vHIP) and basolateral amygdala (AMY). Here, we demonstrate that glutamatergic vHIP afferents to NAc regulate susceptibility to chronic social defeat stress (CSDS). We observe reduced activity in vHIP in mice resilient to CSDS. Furthermore, attenuation of vHIP-NAc transmission by optogenetic induction of long-term depression is pro-resilient, whereas acute enhancement of this input is pro-susceptible. This effect is specific to vHIP afferents to the NAc, as optogenetic stimulation of either mPFC or AMY afferents to the NAc is pro-resilient. These data indicate that vHIP afferents to NAc uniquely regulate susceptibility to CSDS, highlighting an important, novel circuit-specific mechanism in depression.

## Abstract (web summary)

Enhanced glutamatergic transmission in the nucleus accumbens (NAc) has been implicated in the pathophysiology of depression, yet the underlying source is not known. Here, the authors demonstrate a unique role for ventral hippocampal-NAc glutamatergic projections in regulating depression-like behaviour.

## Graphical abstract

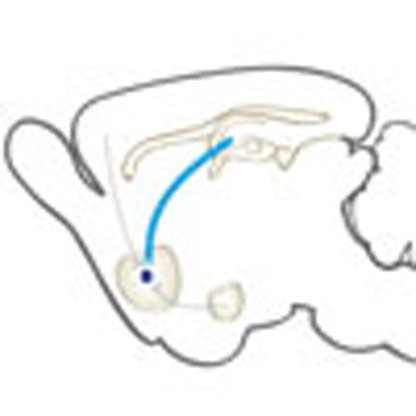

Alterations in nucleus accumbens (NAc), a region critical for reward and motivation, are implicated in the pathophysiology of depression^1^,^2^,^3^,^4^,^5^,^6^,^7^. Glutamatergic transmission in medial NAc is increased in mice exhibiting depression-like behaviour after chronic stress^3^,^4^,^5^. However, the role of presynaptic changes in driving stress-induced postsynaptic adaptations in NAc medium spiny neurons (MSNs) is not well understood. The NAc integrates cortico-limbic afferents with dopaminergic modulation from the ventral tegmental area^8^,^9^. Integration occurs at the cellular level, with individual NAc MSNs receiving convergent glutamatergic projections from ventral hippocampus (vHIP), medial prefrontal cortex (mPFC) and basolateral amygdala (AMY), in addition to other regions such as the thalamus^8^,^10^,^11^,^12^,^13^. Complex competitive interactions between inputs gate postsynaptic MSN responses to afferent activation to direct the finely tuned integration of executive control from mPFC, conditioned associations and emotion from AMY and contextual, spatial and emotion-related inputs from vHIP to orchestrate adaptive motivated behaviour^8^,^9^,^14^,^15^.

mPFC activity is reduced in depressive-like states in rodents, and optogenetic activation of mPFC, a manipulation that, among other actions, increases glutamate release in NAc, is antidepressant^16^,^17^,^18^. These findings are paradoxical in view of observed increases in mini EPSC frequency^4^ and AMPA current^3^ in NAc MSNs of stress-susceptible mice, which point to stress-induced facilitation of both pre- and postsynaptic mechanisms of glutamate transmission. This raises the question of whether chronic stress may produce pathway-specific alterations, with distinct glutamatergic inputs exerting opposing effects on depression-like behaviour^19^,^20^,^21^,^22^. To examine this possibility, we investigated regulation of vHIP-NAc, mPFC-NAc and AMY-NAc projections by chronic social defeat stress (CSDS), a validated mouse model of depression^1^,^2^. Examining immediate early gene (IEG) expression as an indicator of neuronal activity and electrophysiological correlates of presynaptic neurotransmitter release, we report afferent-specific CSDS-induced adaptations in vHIP and mPFC. Using optogenetic manipulations to bidirectionally control afferent-specific synaptic function, we demonstrate a unique role for vHIP-NAc in driving depression-like behavioural phenotypes.

## Results

### CSDS oppositely regulates activity of vHIP and mPFC

**Figure 1:**
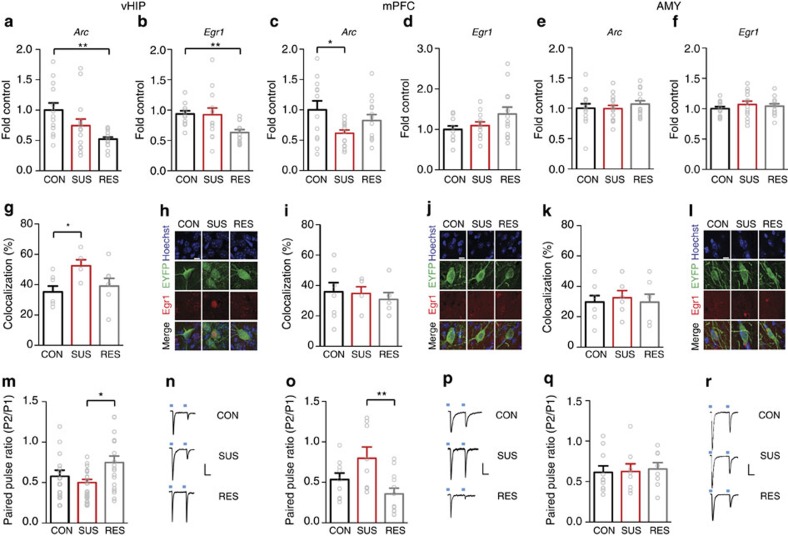
Opposing effects of CSDS on vHIP and mPFC IEG expression and synaptic function. mRNA expression of two immediate early genes in vHIP of control (CON), susceptible (SUS) and resilient (RES) mice. (**a**) *Arc* (F_2,40_=6.485, *P*<0.01, *post hoc* **P<0.01, *n*=13,15,15) and (**b**) *Egr1* (F_2,36_=5.415, *P*<0.01, *post hoc* **P<0.01, *n*=12,14,13) were decreased in vHIP of RES mice 48 h post-defeat. In contrast, (**c**) *Arc* expression was decreased in mPFC of SUS mice (F_2,37_=3.431, *P*<0.05, *post hoc* CON versus SUS *P<0.05, *n*=12,14,14). (**d**) *Egr1* expression was not significantly different (*n*=11,13,12). Neither *Arc* (**e**) nor *Egr1* (**f**) expression was regulated by defeat in AMY (*n*=14,15,15). A greater percentage of EYFP retrogradely labelled NAc-projecting neurons were EGR1 positive in vHIP (**g**) of SUS mice compared to controls (F_2,16_=3.618, *P*<0.05, *post hoc* *P<0.05, *n*=7,5,7). EGR1 levels in mPFC (**i**) and AMY (**k**) in NAc-projecting neurons were not significantly regulated by defeat. Representative images show Hoecsht nuclear stain (blue) in EYFP labelled EGR1 (red) staining in NAc-projecting neurons in vHIP (**h**), mPFC (**j**) and AMY (**l**) labelled by retrograde AAV2/5–CaMKIIa–EYFP injected into NAc (scale bar 10 μm). In RES relative to SUS mice, paired-pulse ratios (P_2_/P_1_; 100 ms interpulse interval) of optically evoked EPSCs in NAc shell MSNs were increased from (**m**) vHIP (F_2,50_=4.023, *P*<0.05, *post hoc* *P<0.05, *n*=14,20,19 cells from *n*=6,7,6 mice) and decreased from (**o**) mPFC (F_2,26_=5.459, *P*<0.01, *post hoc* **P<0.01, *n*=9,8,12 cells from *n*=5,4,7 mice). Paired-pulse ratios from AMY (q) were not regulated by defeat (*n*=10,8,8 cells from *n*=4,3,4 mice). Representative EPSCs evoked by optical stimulation of vHIP (**n**), mPFC (**p**) and AMY (**r**) ChR2-expressing terminals. Vertical scale bar (**n**) 80 pA, (p,r) 40 pA, horizontal scale bar 40 ms. Blue squares indicate light pulse delivery. One-way-ANOVAs with Bonferroni *post hoc* tests. Grubb’s test was used to detect and remove statistical outliers in panels a-f. Error bars represent s.e.m.

We examined mRNA expression of two IEGs, *Arc* and *Egr1*, in mPFC, vHIP and AMY of susceptible and resilient mice versus stress-naive controls to assess basal activation of these brain regions 48 h after CSDS. Mice were determined to be susceptible or resilient based on their social interaction ratio (time in interaction zone with social target/time with target absent) in a social interaction test 24 h after CSDS (susceptible ratio<1; resilient ratio >1; Supplementary Fig. 1). This designation has been highly validated in previous studies and shown to correlate with other depression-related, but not anxiety-related, behavioural abnormalities^1^,^2^,^3^. Both *Arc* and *Egr1* transcript levels were reduced in vHIP of resilient mice versus controls, whereas *Arc*, but not *Egr1*, was decreased in mPFC of susceptible mice and neither transcript was altered in AMY (Fig. 1a–f). To identify vHIP, mPFC and AMY neurons projecting to NAc, we injected the retrograding AAV2/5–CaMKIIa–EYFP virus into NAc before CSDS. Double-immunofluorescence labelling revealed increased EGR1-positive EYFP-labelled vHIP neurons in susceptible mice (Fig. 1g). No significant effects were observed in NAc-projecting mPFC and AMY neurons (Fig. 1i,k). ARC protein levels are too low under these conditions to perform the equivalent analysis. Reduced IEG expression in these regions of resilient versus susceptible mice suggests that decreased vHIP activity might be pro-resilient, whereas decreased mPFC activity is pro-susceptible.

To assess afferent-specific glutamatergic synaptic transmission in NAc, mice were injected with (non-retrograding) AAV5-CaMKIIa–ChR2–EYFP virus in vHIP, mPFC or AMY (Supplementary Fig. 2). The CaMKIIa promoter targets viral infection to glutamatergic neurons within the injected brain regions (see Methods), and we confirmed that optically induced currents are blocked completely by glutamate receptor antagonists (Supplementary Fig. 2c). Six weeks later, when robust transgene expression was seen in nerve terminals in NAc, mice were exposed to CSDS. Using whole-cell patch-clamp in brain slices, optogenetically stimulated paired-pulse responses were recorded in medial NAc from control, susceptible and resilient mice beginning 48 h after CSDS. The resulting ratio of excitatory postsynaptic current (EPSC) amplitudes to paired stimulations is indicative of vesicle release probabilities and suggestive of changes in glutamate release^23^. Paired-pulse ratios (PPRs) of vHIP afferent-stimulated responses in NAc were increased in resilient relative to susceptible mice, suggesting a potential decreased probability of glutamate release (Fig. 1m). In contrast, PPRs of mPFC afferent-stimulated responses were decreased in resilient animals relative to susceptible mice (Fig. 1o). PPRs of AMY afferent-stimulated responses were not regulated by CSDS (Fig. 1q). These findings suggest that glutamate release at vHIP-NAc synapses may be decreased in resilient mice, whereas mPFC-NAc synaptic glutamate release is increased in resilient mice.

### LTD attenuation of vHIP-NAc synaptic transmission is pro-resilient

**Figure 2:**
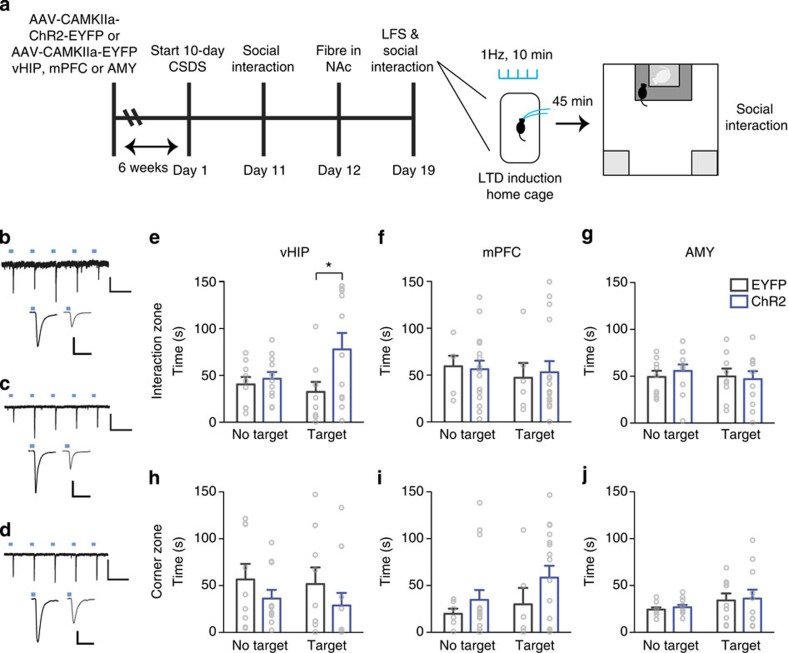
LTD at vHIP-NAc induces resilience to CSDS. (**a**). Schematic of experimental time-course. Representative traces validating that 1 Hz stimulation of ChR2-expressing vHIP (**b**) (scale bar 200 pA, 1 s), mPFC (**c**) (scale bar 50 pA, 1 s) and AMY (**d**) (scale bar 200 pA, 1 s) terminals reliably evoke EPSCs with temporal fidelity in NAc MSNs (upper panels). 10 min, 1 Hz stimulation (low frequency stimulation; LFS) induces LTD of evoked EPSCs measured 45 min after LFS (**b**–**d**, lower panels; scale bars 80 pA, 50 ms) 45 min post-LFS (grey traces) compared to pre-LFS baselines (black traces). Blue squares indicate light pulse delivery. (**e**) *In vivo*, LFS of vHIP-NAc synapses in defeated mice increased time spent in the interaction zone in the presence of a target (F_1,18_=5.274 interaction effect, *P*<0.05, *post hoc* *P<0.05, *n*=9,11) but did not alter time spent in corners (**h**). Stimulation of mPFC-NAc (**f**; *n*=6, 16) or AMY-NAc synapses (**g**; *n*=9,11) had no effect on time spent in interaction or corner zones (**i**, **j**). Two-way repeated measures ANOVA with Bonferroni *post hoc* tests. ‘EYFP’ and ‘ChR2’ denote AAV5-CaMKIIa–EYFP and AAV5-CaMKIIa–ChR2–EYFP, respectively. ‘No target’ and ‘Target’ indicate absence or presence of a target mouse during testing. Error bars represent s.e.m.

To determine whether decreasing glutamatergic synaptic transmission at vHIP-NAc synapses could promote resilience in previously defeated mice, we injected AAV5-CaMKIIa–ChR2–EYFP or AAV5–CaMKIIa–EYFP into vHIP and 6 weeks later exposed mice to CSDS. Optic fibres targeting medial NAc were then surgically implanted, and optogenetic low frequency stimulation (LFS; 473 nm, 10 min, 1 Hz, 4 ms pulse width) of ChR2-infected terminals in NAc was used to induce long-term depression (LTD) of MSN EPSCs. One week after surgery, we delivered LFS to reduce vHIP-NAc synaptic transmission 45 min before social interaction testing (Fig. 2a); LTD-like responses were electrophysiologically validated by optogenetic terminal stimulation of inputs in brain slices and *in vivo* (Fig 2b-d; Supplementary Fig. 3a-c) as previously described^24^. vHIP ChR2-infected mice spent more time interacting with a target mouse in comparison with EYFP-infected controls (Fig. 2e) and similar to what is normally observed in resilient mice indicating a pro-resilient effect. The same manipulations did not affect social interaction in mice injected with AAV5-CaMKIIa–ChR2–EYFP in mPFC or AMY (Fig. 2f,g). vHIP, mPFC or AMY injected stress-naive controls were unaffected by optical stimulations (Supplementary Fig. 3).

### Enhancement of vHIP-NAc synaptic transmission is pro-susceptible

**Figure 3:**
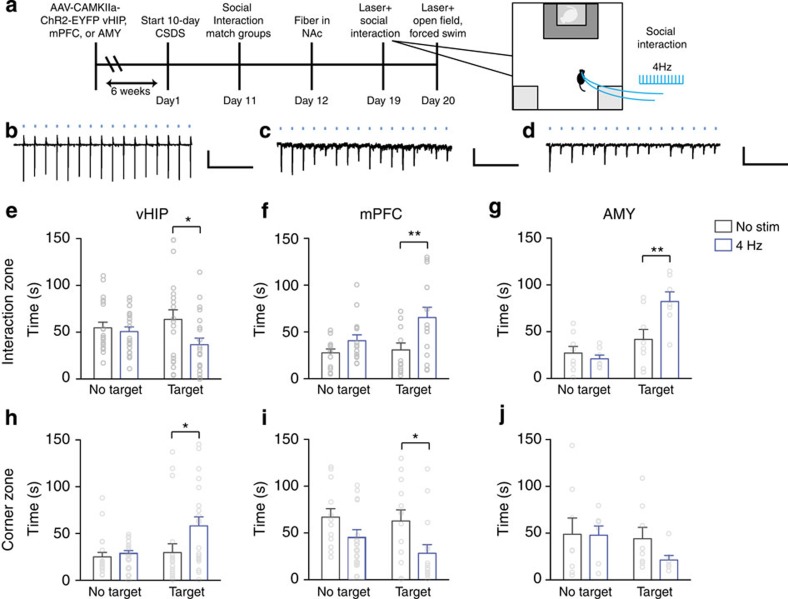
Acute stimulation of vHIP versus mPFC or AMY terminals in NAc promotes opposite behavioral responses to CSDS. (**a**). Schematic of experimental time course. Representative traces validating that 4 Hz stimulation of vHIP (**b**) (scale bar 200 pA, 1 s), mPFC (**c**) (scale bar 50 pA, 1 s) and AMY (**d**) (scale bar 50 pA, 1 s) terminals in NAc slices evokes EPSCs. Blue squares indicate light pulse delivery. *In vivo*, 4 Hz stimulation of vHIP terminals in NAc shell (**e**) reduced time spent interacting with a target mouse (F_1,39_=4.274 interaction effect, *P*<0.05, *post hoc* *P<0.05, *n*=20,21) and (**h**) increased time spent in corners (F_1,39_=6.560 target effect, F_1,39_=4.271, stimulation effect, *P*<0.05, *post hoc* *P<0.05, *n*=20,21). Stimulation of mPFC terminals in NAc (**f**) increased time spent interacting with a target mouse (F_1,26_=9.174 stimulation effect, *P*<0.01, *post hoc* **P<0.01, *n*=13,15) and (**i**) decreased time spent in corners (F_1,26_=7.735, *P*<0.01, *post hoc* *P<0.05, *n*=13,15). Stimulation of AMY axons in NAc (**g**) increased time spent interacting with a target mouse (F_1,14_=5.4284 interaction effect, *P*<0.01, *post hoc* **P<0.01, *n*=8) but (**j**) did not affect time spent in corners. Two-way repeated-measures ANOVAs with Bonferroni *post hoc* tests. ‘No stim’ and ‘4 Hz’ denote non-stimulated controls versus 4 Hz stimulated mice. ‘No target’ and ‘Target’ indicate absence or presence of a target mouse during testing. Error bars represent s.e.m.

To examine whether conversely increasing glutamate synaptic transmission in NAc could increase stress susceptibility, vHIP terminals in medial NAc were acutely stimulated to increase glutamate release at vHIP-NAc synapses during social interaction testing following CSDS (Fig. 3a; fidelity of acutely evoked EPSCs was validated in brain slices Fig. 3b–d; and *in vivo*,i> Supplementary Fig. 4a–c). Mice receiving acute optical stimulation (4 Hz, 5 ms) during social interaction testing spent less time interacting with a target animal and increased time in corner zones compared with non-stimulated controls (Fig. 3e,h). To assess whether increasing glutamate release selectively at mPFC-NAc synapses could increase resilience to stress, similar to the effect of global mPFC stimulation^16^, we acutely stimulated mPFC terminals in medial NAc. Mice receiving acute stimulation of mPFC terminals in NAc spent more time interacting with a target mouse and less time in the corner zones (Fig. 3f,i). Acutely stimulated mice that were injected intra-AMY with AAV5-CAMKIIa–ChR2–EYFP spent more time interacting with a target mouse (Fig. 3g,j), similar to the effect of mPFC stimulation. As an additional control, we confirmed that light stimulation of EYFP-injected control mice and non-stimulated ChR2-injected mice exhibited equivalent behavioural responses (Supplementary Fig. 5)

Acute stimulation of each of the three distinct NAc afferents, vHIP, mPFC or AMY, did not affect anxiety-like behaviour in an open field test, indicating that the effects on social interaction are not confounded by effects on anxiety (Supplementary Fig. 4d–f). Moreover, acute vHIP terminal stimulation increased immobility in the forced swim test, confirming a pro-depressant behavioural effect (Supplementary Fig. 4g). In contrast, acute AMY terminal stimulation decreased immobility, whereas mPFC terminal stimulation had no effect (Supplementary Fig. 4h,i). Stimulation of each of the three afferents increased locomotor activity in the open field test (Supplementary Fig. 4j–l). Effects of acute stimulation on social-avoidance behaviour require previous stress exposure: manipulations in stress-naive-mice did not alter social interaction. Of note, AMY stimulation decreased exploration of the empty interaction zone, but time spent interacting with a target mouse was not affected (Supplementary Fig. 6).

## Discussion

NAc MSNs receive dense glutamatergic projections from vHIP, mPFC and AMY, among other regions. Here, we present data suggesting that vHIP-NAc synaptic transmission is selectively increased in mice susceptible to CSDS, and demonstrate that *in vivo* optogenetic manipulations of vHIP-NAc synaptic transmission bidirectionally regulate depression-like behaviours. In contrast, our findings suggest that mPFC-NAc synaptic transmission is decreased in susceptible animals, and show that increasing either mPFC or AMY synaptic transmission by acute stimulation *in vivo* promotes resilience.

The mechanism of these distinct pathway-specific adaptations is unclear. Opposing effects of vHIP versus mPFC and AMY synaptic transmission could reflect the relative strengths of the examined inputs; vHIP projections to NAc shell are denser than those arising from mPFC or AMY^12^. Also, although all of these inputs are rewarding in stress-naive mice, stronger stimulations of mPFC and AMY are necessary to achieve this rewarding effect^12^,^25^,^26^. Previous work has demonstrated that blockade of AMPA receptors in NAc increases resilience to stress^3^. This finding was difficult to integrate into a circuit-level understanding given: (1) established findings that activity of mPFC, a major source of glutamatergic innervation in the NAc, is reduced in depression and (2) the therapeutic efficacy of mPFC stimulation^16^,^27^,^28^,^29^,^30^. The results of the present study suggest that the therapeutic effect of NAc AMPA blockade is mediated by vHIP-NAc glutamate synapses. In addition, it is possible that these afferents differentially target the two MSN subtypes (Drd1- versus Drd2-expressing) to mediate opposing behavioural effects^26^,^31^,^32^ and that stress may shift the cell-type-specific balance of connectivity in this circuit, a possibility that warrants direct examination.

The uniquely pro-depressant role of vHIP-NAc glutamate projections in chronically stressed mice opposes established roles for these projections in facilitating reward in stress-naive mice^12^,^25^,^26^. A recent paper examined the regulation of plasticity in the vHIP-NAc pathway in another rodent depression model (learned helpless rats) by electrically stimulating the fimbria and recording cell firing in the NAc^33^. Intriguingly, this study reported that a stimulation that produces long-term potentiation in control rats induced LTD in learned helpless rats. Synaptic plasticity at glutamatergic synapses is tightly regulated by homeostatic and metaplastic processes. Increased activity at glutamatergic synapses can facilitate the later induction of LTD^34^,^35^,^36^. Although methodological and species differences between the two studies limit the ability to draw direct comparisons, it is interesting to speculate whether this earlier finding might also suggest that glutamatergic synaptic transmission in the vHIP-NAc pathway is potentiated in learned helpless rats, thus allowing later electrical stimulation to induce LTD. While human structural imaging studies have reported decreased hippocampal volume in depressed subjects, functional imaging studies report indicators of increased hippocampal glutamate associated with familial risk of depression, increased hippocampal activation during negative information processing and an association between decreased hippocampal metabolism and antidepressant responses^27^,^28^,^37^,^38^. The mechanism of stress-dependent regulation of vHIP glutamate projections may reflect an interaction with alterations in dopaminergic input to NAc. Ventral tegmental area dopamine neuron burst firing is increased in susceptible mice^1^,^39^,^40^, and phasic dopamine release in NAc has been shown to facilitate vHIP-NAc inputs^14^. Interestingly, vHIP-NAc stimulation may also increase VTA dopamine neuron population activity^41^.

Our findings highlight a complex afferent-specific role for glutamatergic signalling in NAc in depression-associated behaviours. Chronic stress may shift the balance away from mPFC towards vHIP control, creating a reinforcing loop further enhancing vHIP-NAc synaptic transmission. Circuit-level therapeutic interventions that attenuate presynaptic vHIP over-activation in addition to targeting postsynaptic alterations in NAc may constitute more effective treatment strategies.

## Methods

### Experimental animals

Male 6–8 week-old C57BL/6 J mice, and 6-month-old CD1 retired breeders, were maintained on a 12-h light–dark cycle (lights on at 0700 hours) at 22–25 °C with *ad libitum* access to food and water. C57 mice were housed 5 per cage except following defeat experiments and after fiber implantation surgery at which point mice were singly housed. All experiments were conducted in accordance with the guidelines of the Institutional Animal Care and Use Committee at Icahn School of Medicine at Mount Sinai. All behavioural testing occurred during the animals’ light cycle. Experimenter was blinded to experimental group and order of testing was counterbalanced during behavioural experiments and across days for electrophysiology experiments. For *in vivo* LTD experiments (Fig. 2), animals were randomized within cages before surgery. For acute stimulation experiments (Fig. 3), groups were matched on post-defeat social interaction scores to generate two groups with equivalent means and variance.

### RNA isolation and qPCR

Mice were killed 48 h after CSDS (see below), brains were removed, coronally sliced and mPFC, vHIP and AMY tissue was rapidly dissected and frozen on dry ice. RNA isolation, qPCR and data analyses were performed as described^1^. Briefly, RNA was isolated with TriZol reagent (Invitrogen) and purified with RNAeasy micro kits from Qiagen. All RNA samples were determined to have 260/280 and 260/230 values ≥1.8. Reverse transcription was performed using iScript (BioRad). qPCR using SYBR green (Quanta) was carried out with an Applied Biosystems 7900HT RT–PCR system with the following cycle parameters: 2 min at 95 °C; 40 cycles of 95 °C for 15 s, 59 °C for 30 s, 72 °C for 33 s; and graded heating to 95 °C to generate dissociation curves for confirmation of single-PCR products. Data were analysed by comparing C(t) values of conditions tested (control versus susceptible or resilient mice) using the ΔΔC(t) method^42^. qPCR primers: *Egr1* FWD: 5′-GAGGAAGTTTGCCAGGAGTG-3′, *Egr1* REV: 5′-GAGTAGGAAGTGGGCACAGG-3′; *Arc* FWD: 5′-GAAGTGGTGGGAGTTCAAGC-3′, *Arc* REV: 5′-TCCTCAGCGTCCACATACAG-3′.

### Immunohistochemistry

Forty-eight hours after CSDS (see below), mice were anaesthetized with chloral hydrate and transcardially perfused with 0.1M PBS followed by 4% paraformaldehyde in PBS. Brains were postfixed overnight and then cryopreserved in 30% sucrose. Brains were sectioned on a freezing microtome at 35 μm. Sections were rinsed three times in PBS and blocked in 4% normal donkey serum (NDS) with 0.5% Triton-X in PBS (PBST) for 2 h at room temperature. Sections were then incubated in primary antibodies overnight at 4 °C (chicken anti-GFP, #AB13970, Abcam, 1:500 and rabbit anti-Egr1, #4154 S, Cell Signaling, 1:1,000) in PBST. Sections were then washed three times in PBS and incubated with secondary antibodies (donkey anti-rabbit Cy3, donkey anti-chicken Alexa Fluor 488; 1:500, Jackson ImmunoResearch) for 2 h at room temperature. Sections were washed three times in PBS. Hoechst stain (Invitrogen) was added to the final wash for 25 min. Sections were washed one final time before mounting and coverslipping with ProLong Gold (Invitrogen).

### Stereotaxic surgery and viral vectors

Adenoassociated virus (AAV) vectors expressing channelrhodopsin-2 (ChR2) fused with enhanced yellow fluorescent protein (EYFP) under the control of the CaMKIIa promoter (AAV5–CaMKIIa–ChR2–EYFP) were used for optical activation of glutamatergic afferent terminals of NAc (mPFC, vHIP and AMY). CaMKIIa expression is specific to excitatory glutamatergic neurons^43^, and the use of this promoter in viral vectors has been shown to target such neurons^12^,^44^,^45^. AAV5–CaMKIIa–ChR2–EYFP and AAV5–CaMKIIa–EYFP were purchased from the University of North Carolina Vector Core. To label neurons projecting to NAc, retrograding AAV2/5–CaMKIIa–EYFP purchased from University of Pennsylvania Viral Core was infused into NAc. (Note that AAV2/5 represents a different serotype than the AAV5 vector, even though the UPenn Core labels it as ‘AAV5.’) For stereotaxic surgeries, mice were anaesthetised with a mixture of ketamine (100 mg kg^−1^) and xylazine (10 mg kg^−1^) and positioned in a small-animal stereotaxic instrument (Kopf Instruments). The skull surface was exposed and 33-gauge syringe needles (Hamilton) were used to bilaterally infuse 0.5 μl AAV5–CaMKIIa–ChR2–EYFP or control vector AAV5–CaMKIIa–EYFP at a rate of 0.1 μl min^−1^ into mPFC (bregma coordinates: anterior/posterior, 1.7 mm; medial/lateral, 0.75 mm; dorsal/ventral, −2.5 mm; 15° angle; targeting infralimbic prefrontal cortex), vHIP (bregma coordinates: anterior/posterior, −3.7 mm; medial/lateral, 3 mm; dorsal/ventral, −4.8 mm; 0° angle; targeting ventral subciulum) or AMY (bregma coordinates: anterior/posterior, −1.6 mm; medial/lateral, −3.1 mm; dorsal/ventral, −4.9 mm; 0° angle; targeting basolateral amygdala). The targeted regions all project to the NAc shell region targeted by our *in vivo* and *in vitro* manipulations^12^. Chronically implantable optic fibers constructed with 200 μm core 0.22 NA optic fiber (Thor Labs) threaded through ceramic zirconia ferrules (Precision Fibre Products) were implanted in NAc shell (bregma coordinates: anterior/posterior, 1.3 mm; medial/lateral, 1.6 mm; dorsal/ventral, −4.4 mm; 10° angle) and secured with dental cement (Grip Cement Dentsply). For robust viral expression in projection terminals of NAc shell, viral infusions occurred a minimum of 6 weeks before stimulation.

### *In vitro* optogenetic patch-clamp electrophysiology

All recordings were conducted blind to the experimental conditions 2–28 days after CSDS. Whole-cell recordings were obtained from NAc MSNs in acute brain slices from mice that had been stereotaxically injected with AAV5–CaMKIIa–ChR2–EYFP into mPFC, vHIP or AMY. Eight to 12 weeks after viral surgery, mice were anaesthetized with isofluorane and perfused 1 min with ice-cold aCSF (128 mM NaCl, 3 mM KCl, 1.25 mM NaH_2_PO_4_, 10 mM D-glucose, 24 mM NaHCO_3_, 2 mM CaCl_2_ and 2 mM MgCl_2_; oxygenated with 95% O_2_ and 5% CO_2_, pH 7.4, 295–305 mOsm). Acute brain slices (200 μm) containing NAc shell were cut using a microslicer (DTK-1000, Ted Pella) in cold sucrose aCSF (254 mM sucrose, 3 mM KCl, 1.25 mM NaH_2_PO_4_, 10 mM D-glucose, 24 mM NaHCO_3_, 2 mM CaCl2 and 2 mM MgCl_2_; oxygenated with 95% O_2_ and 5% CO_2_, pH 7.4, 295–305 mOsm). Slices were incubated in aCSF for 1 h at 32 °C and then returned to room temperature for a minimum of 1 h before recording. Patch pipettes (3–5 mΩ) for whole-cell voltage-clamp recordings were filled with internal solution (115 mM potassium gluconate, 20 mM KCl, 1.5 mM MgCl_2_, 10 mM phosphocreatine, 10 mM HEPES, 2 mM magnesium ATP and 0.5 mM GTP; pH 7.2, 285 mOsm). Slices were continuously perfused with room temperature aCSF with 50 μm picrotoxin (Sigma; flow rate=2.5 ml min^–1^). Slices were examined to confirm robust viral expression at injection site and in NAc shell before proceeding. MSNs were voltage clamped at −80 mV, and ChR2-expressing terminals were stimulated by blue light pulses generated by a stimulator (Agilent Technologies) and laser (50 mW; OEM laser systems) connected to a 200 μm core optic fiber positioned above the slice aimed at the recorded cell. Optically evoked EPSCs were obtained every 20 s with paired pulses of 473 nm light (30 mW, 0.5–3 ms; 100 ms interpulse interval) and recorded in voltage-clamp mode using the Multiclamp 700B amplifier. Data were acquired using pClamp 10 software (Molecular Devices). Series and access resistance were monitored during the experiments and signals were bessel filtered at 1.8 kHz (gain10). Control experiments confirmed the stability of the PPR with the range of pulse widths (0.5 ms–3 s) and within a range of laser intensities (10–30 mW) although variability was observed at very low intensities (5 mW; Supplementary Fig. 7).

### *In vivo* optogenetic electrophysiology

*In vivo* extracellular field recordings from NAc of anaesthetised intact mice were conducted following published methods.^39^ Mice were anaesthetised with 8% chloral hydrate (400 mg kg^−1^, i.p.). The skull was exposed and the area above the NAc was removed to lower an optrode consisting of a glass electrode (15–20 MΩ filled with 2 mM NaCl) tightly glued to an optic fiber. NAc coordinates were measured from bregma and were as follows (in mm): anteroposterior, 1.7–0.86 mm; mediolateral, 0–1.6 mm; and dorsoventral, 3.3–4.7 mm. Using a DP-311 Differential Amplifier (Warner Instruments), extracellular optically evoked field responses were amplified (× 1,000) and filtered (0.3–1 kHz band pass). Optically evoked voltage data were acquired using an Axon Digidata Data Acquisition System (Molecular Devices), sampled using 16-bit resolution at 32 kHz, and stored using pCLAMP for further offline analysis.

### *In vivo* optogenetic stimulation

Custom optical fiber patch cords attached to implanted fibers^46^ with ceramic zirconia sleeves (Precision Fiber Products) were connected using an FC/PC adaptor to a 473-nm blue laser diode (OEM Laser Systems; 100 mW) and a stimulator to generate blue light pulses. LFS to induce LTD consisted of 10 min, 1 Hz, 4 ms pulse width 15–20 mW, 473 nm stimulation 45 min before social interaction testing. In separate experiments, synaptic transmission was acutely increased during behavioural testing using 4 Hz, 5 ms pulse width, 15–20 mW 473 nm stimulation. This stimulation protocol has previously been shown to reliably modulate synaptic transmission by ChR2-expressing glutamatergic terminals in NAc^12^. Comparison of EYFP-injected stimulated controls and ChR2 injected non-stimulated controls showed no differences in social interaction behaviour (Supplementary Fig. 5).

### CSDS and social interaction

All experiments utilized an established CSDS protocol to induce depressive-like behaviours in mice^2^. C57BL/6 J mice were subjected to 10 daily, 5-min defeats by a novel CD1 aggressor mouse and were then housed across a plexiglass divider to allow for sensory contact. Control mice were housed in cages separated from other control mice by a plexiglass divider and were rotated to a different cage daily. Resilient and susceptible mice were identified by their respective preference for, or avoidance of, interaction with a novel mouse after 10 days of defeat in a social interaction test. *In vivo* optogenetic experiments used the entire population of defeated mice and did not specifically select for resilient or susceptible to facilitate the detection of bidirectional shifts in social interaction behaviour.

Social-avoidance behaviour was assessed with a novel CD1 mouse in a two-stage social-interaction test. In the first 2.5-min test (no target), the experimental mouse was allowed to freely explore an arena (44 × 44 cm) containing a plexiglass and wire mesh enclosure (10 × 6 cm) against one wall of the arena. In the second 2.5-min test (target), the experimental mouse was returned to the arena with a novel CD1 mouse enclosed in the plexiglass wire mesh cage. Time spent in the ‘interaction zone’ (14 × 26 cm) surrounding the plexiglass wire mesh cage, ‘corner zones’ (10 × 10 cm) and ‘distance travelled’ within the arena was measured by video tracking software (Ethovision 5.0, Noldus).

### Open field

Exploration of an open field arena (44 × 44 cm) was assessed during a 5-min test. A video-tracking system (Ethovision 5.0, Noldus) measured locomotor activity, as well as the time spent in the centre (34 × 34 cm) and periphery of the test arena as an index of anxiety.

### Forced swim test

Mice were individually placed in beakers of 25 °C water for 5 mins. Immobility was assessed by a video-tracking system (Ethovision 5.0, Noldus). We utilized a single-forced swim paradigm as extensively used and validated in mice^3^.

### Cellular imaging

One hundred micrometres vibratomed coronal sections containing vHIP, mPFC or AMY, as well as respective NAc, from AAV infected mice, were mounted and coverslipped with ProLong Gold (Invitrogen). Sections were imaged for native EYFP fluorescence (GFP filter) on an Olympus BX51 upright immunofluorescence microscope. Images of viral injection sites (vHIP, mPFC and AMY) were imaged at 4 × objective, and images of afferent axons in NAc were imaged at 10 × objective. All images were obtained at equal gain and exposure and were further processed with NIH Image J. For quantification of immunohistochemistry slides were imaged on a Axio Observer Inverted Microscope (Zeiss) fitted with fluorescent filters. For each animal, sections along the A–P axis of the region of interest were imaged at the indicated magnification (AxioVision imaging software, Zeiss) by an observer blind to condition. Analysis of cell counts and colocalization was performed using ImageJ. Intensity thresholds were adjusted to accurately count moderate- to highly-expressing EGR1+ cells. Density is reported for cell counts to account for variation in region size. Statistical analysis was performed on the average from each animal when multiple sections were imaged and counted. For colocalization analysis, images were acquired on an inverted TCS SP8 laser scanning confocal microscope (Leica DMI 6000) and deconvolved (AutoQuant). Cells were imaged using a 60 × oil immersion lens (numerical aperture 1.4; Leica) and a zoom of 1.0. Images were acquired with a resolution of 512 × 512 (gain=20%), and the line average was set to 4.

### Statistics

No statistical methods were used to predetermine sample sizes but sample sizes are consistent with those reported in previous publications from our laboratory and sufficient for the types of experiments. Variance was similar between groups being statistically compared and data were normally distributed supporting the use of parametric statistics. Some animals or data points were lost in the course of social defeat, due to cage-flooding post-surgery, loss of fiber implant or equipment failure during behavioural testing. Grubb’s test was used to identify and remove significant outliers from qPCR data. qPCR and paired-pulse data were analysed by one-way ANOVA and social interaction data were analysed by two-way repeated measures ANOVA. In all instances, Bonferroni post-hoc tests were used to resolve significant main effects or interactions. In one-way designs all pairwise comparisons were tested and in two-way repeated measures designs the effect of the between subjects variable was compared at each level of the within subjects variable. Two group comparisons (open field and forced swim test) were analysed by unpaired two-tailed Student’s *t*-tests. Significance was set at *P*<0.05 for all tests and only comparisons with *P*<0.05 are explicitly reported. Detailed statistics are provided in figure legends. All data expressed as mean±s.e.m.

## Additional information

**How to cite this article**: Bagot, R.C. *et al*. Ventral Hippocampal Afferents to the Nucleus Accumbens Regulate Susceptibility to Depression. *Nat. Commun*. 6:7062 doi: 10.1038/ncomms8062 (2015).

## Supplementary Material

Supplementary Information Supplementary Figures 1-7
