# Supplementary material for: Ventral hippocampal afferents to the nucleus accumbens regulate susceptibility to depression
Source: Nat Commun. 2015 May 8;6:7062. doi: 10.1038/ncomms8062 (PMC4430111; doi:10.1038/ncomms8062)
Supplement: Supplementary Information — Supplementary Figures 1-7 [file ncomms8062-s1.pdf]

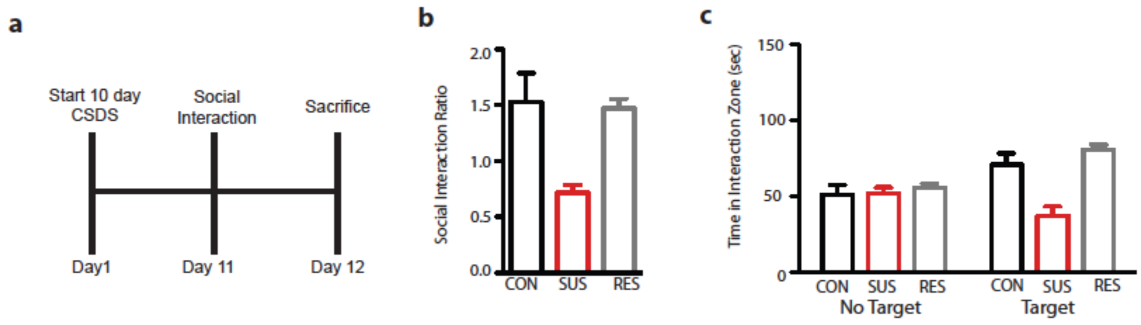

<u>Supplementary Figure 1.</u> Behavioral characterization of control, susceptible and resilient mice. (a) Schematic detailing time-course of chronic social defeat, social interaction testing and tissue collection. (b) To identify susceptible and resilient mice, a social interaction ratio (time in interaction zone with social target/time with target absent) was calculated for each mouse based on (c) behavior in the social interaction test 24 hr after CSDS (susceptible ratio <1; resilient ratio >1).

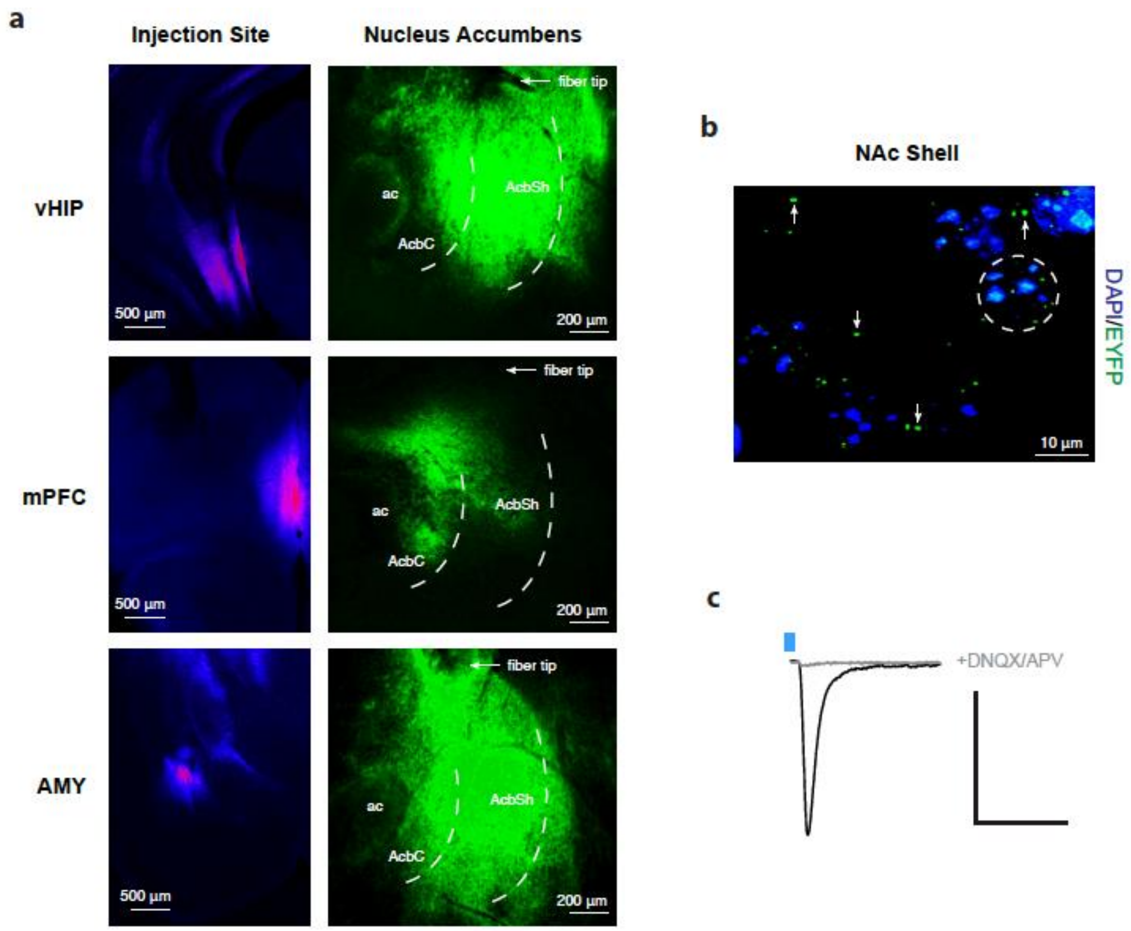

<u>Supplementary Figure 2.</u> Representative coronal sections demonstrating expression of ChR2-EYFP after AAV injection into vHIP, mPFC or AMY. (a) Left panel shows site of virus injection (EYFP in pink). Right panel shows AAV5-CAMKIIa-ChR2-EYFP infected terminals in NAc. All images were captured and processed using NIH Image J. Optic fiber placements are indicated by white arrows. Abbreviations: ac = anterior commissure, AcbC = NAc core, AcbSh = NAc shell. (b) Examples of AAV5-CaMKIIa-ChR2-EYFP infected terminals in NAc are indicated by white arrows. (c) Optogenetic stimulation of AAV5-CaMKIIa-ChR2-EYFP infected terminals in NAc evoked EPSCs in NAc medium spiny neurons *in vitro* that are blocked by AMPA (DNQX 20μm) and NMDA (APV 50 μm) receptor antagonists. Vertical scale bar (n) 80 pA, (p,r) 100 pA, horizontal scale bar 50 ms.

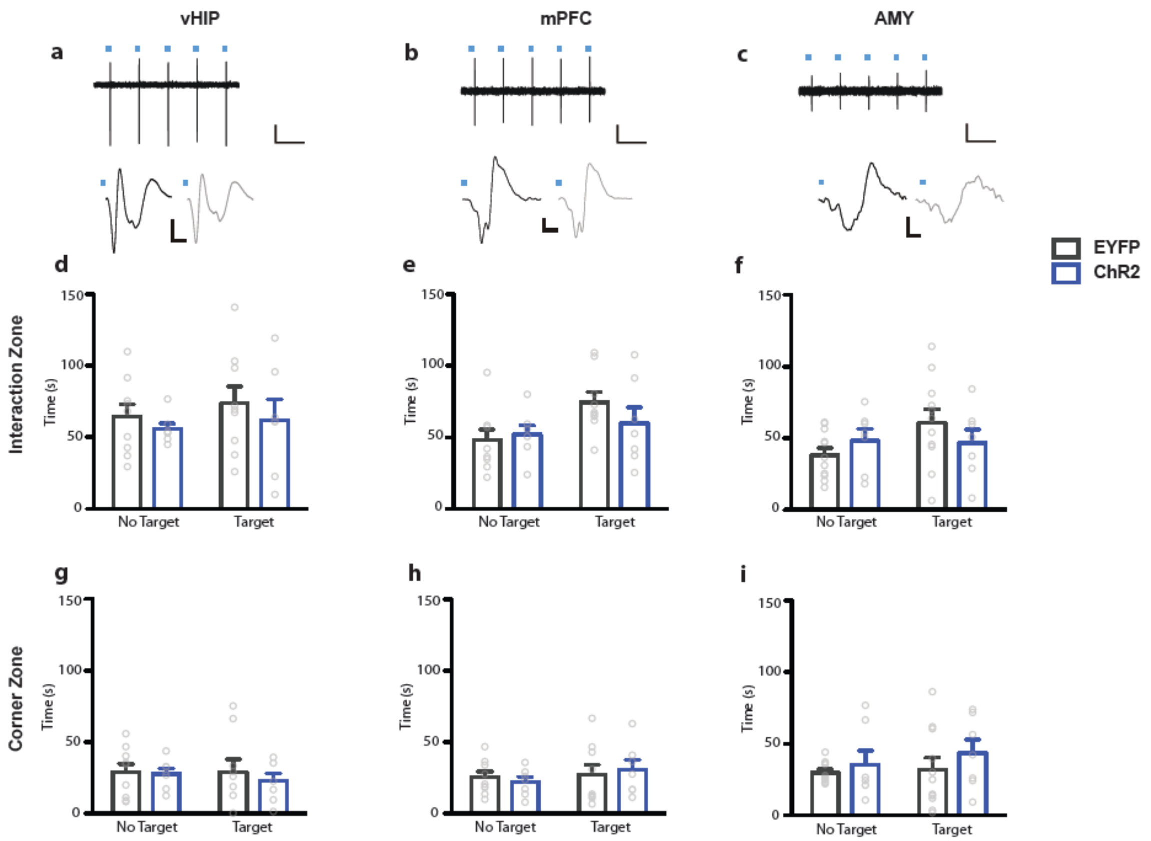

<u>Supplementary Figure 3.</u> LTD at vHIP-NAc, mPFC-NAc or AMY-NAc synapses does not influence social interaction in stress-naïve mice. Representative traces in upper panels validate that 1 Hz stimulation of vHIP- (a; scale bar 0.2 mv, 1 sec), mPFC- (b; scale bar 0.2 mv; 1 sec) and AMY- (c; scale bar 0.1 mv, 1 sec) terminals evoke *in vivo* field responses with temporal fidelity in NAc in anesthetized mice. Lower panels: 10 min, 1 Hz stimulation (low frequency stimulation; LFS) of vHIP (a; scale bar 0.1 mV, 2 ms), mPFC (b; scale bar 0.1 mV, 2 ms) and AMY (c; scale bar 0.05 mV, 2 ms) terminals induce LTD of optically-evoked field responses 45 min post-LFS (grey traces) compared to pre-stimulation baselines (black traces). Blue squares indicate light pulse delivery. *In vivo*, LFS stimulation of vHIP-NAc synapses (n=9,7), mPFC-NAc synapses (n=9,7) or AMY-NAc synapses (n=11,7) in stress-naïve mice did not affect time spent in interaction zone (d-f) or (g) time spent in corners (g-h). Two-way repeated measures ANOVAs. 'EYFP' and 'ChR2' denote AAV5-CaMKIIa–EYFP and AAV5-CaMKIIa-ChR2-EYFP, respectively. 'No target' and 'target' indicate absence or presence of a target mouse during testing.

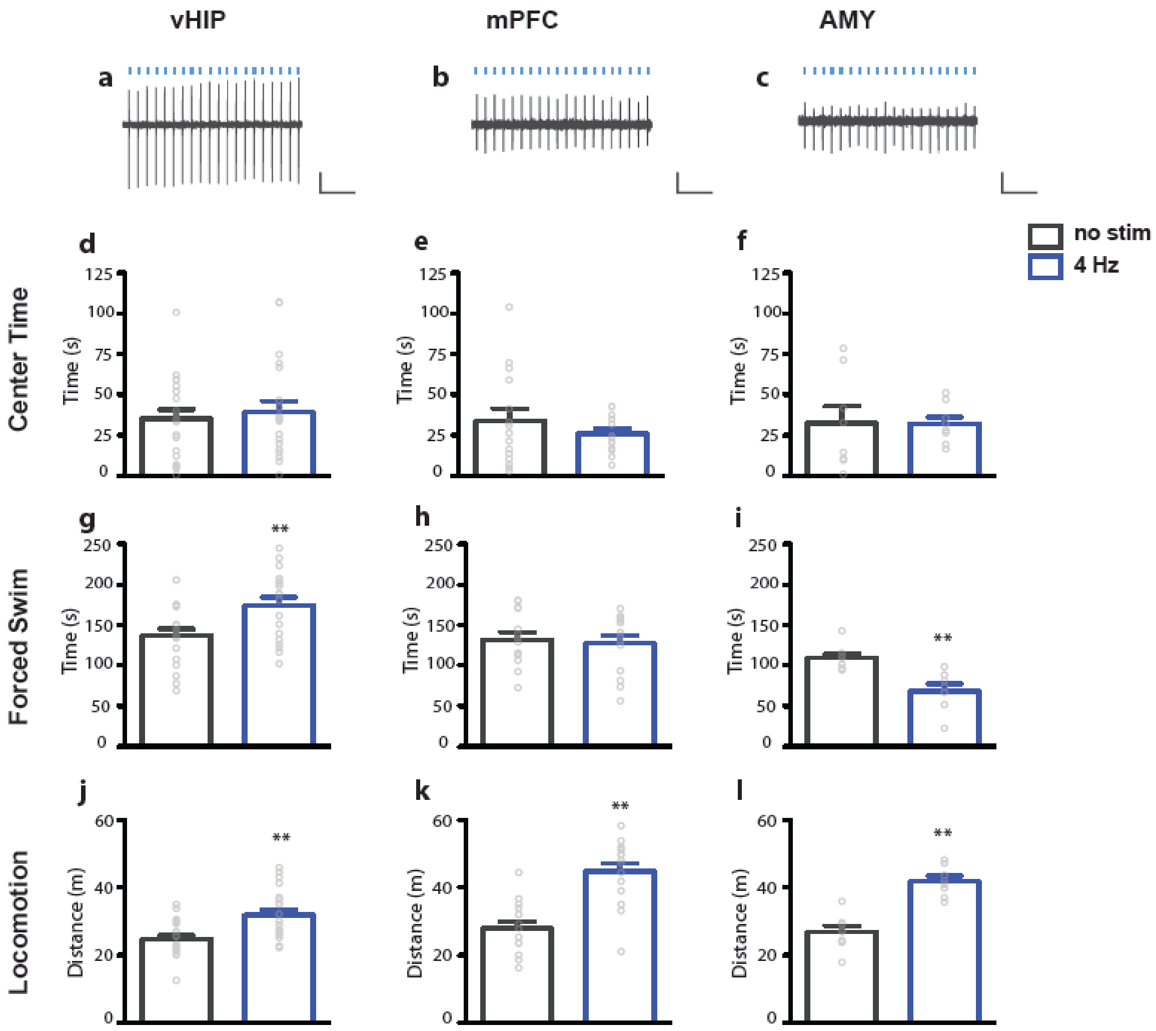

<u>Supplementary Figure 4.</u> Acute stimulation of vHIP, mPFC or AMY terminals in NAc alters FST and locomotor behavior. Representative traces validate that 4 Hz vHIP- (a; scale bar 0.2 mv, 1 sec), mPFC- (b; scale bar 0.2 mv; 1 sec) and AMY- (c; scale bar 0.1 mv, 1 sec) terminals reliably evoke *in vivo* field responses in NAc in anesthetized mice. In previously defeated mice, 4 Hz stimulation of terminals in NAc shell arising from vHIP (d), mPFC (e) or AMY (f) did not alter anxiety-related behavior as measured by time spent in the center of an OFT. In contrast, immobility time in the FST was regulated by such stimulation. Stimulation of vHIP (g) terminals increased time immobile (t=2.93, **p<0.01, n=19), mPFC (h) stimulation had no effect (n=14,15) and AMY (i) stimulation decreased time immobile (t=3.91, **p<0.01, n=8,7). Distance traveled in the OFT was increased by stimulation of terminals from (j) vHIP (t=3.57, **p<0.01, n=20), (k) mPFC (t=5.482642, **p<0.01, n=15,16) or (l) AMY (t=6.14, **p<0.01, n=8). P-values refer to analyses by student's t-tests. 'No stim' and '4 Hz' denote non-stimulated control mice and 4 Hz stimulated mice, respectively.

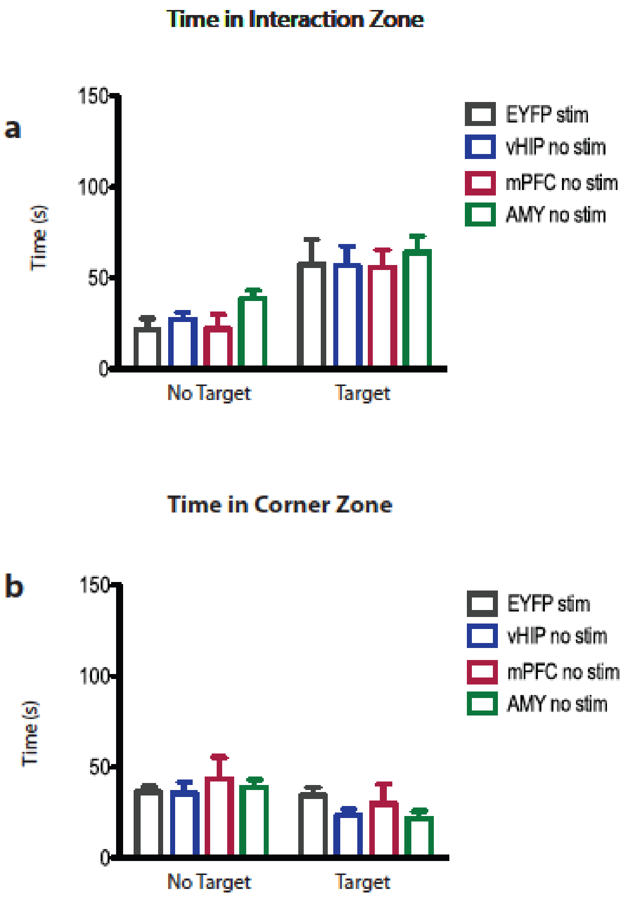

Supplementary Figure 5. Comparison of stimulated and non-stimulated control groups. No effect of stimulation was observed in the absence of ChR2 expression in the social interaction test. EYFP mice receiving 4 Hz stimulation did not differ from non-stimulated mice injected with ChR2 in (a) time spent in interaction zone or (b) time spent in corner zone with or without the social target present.

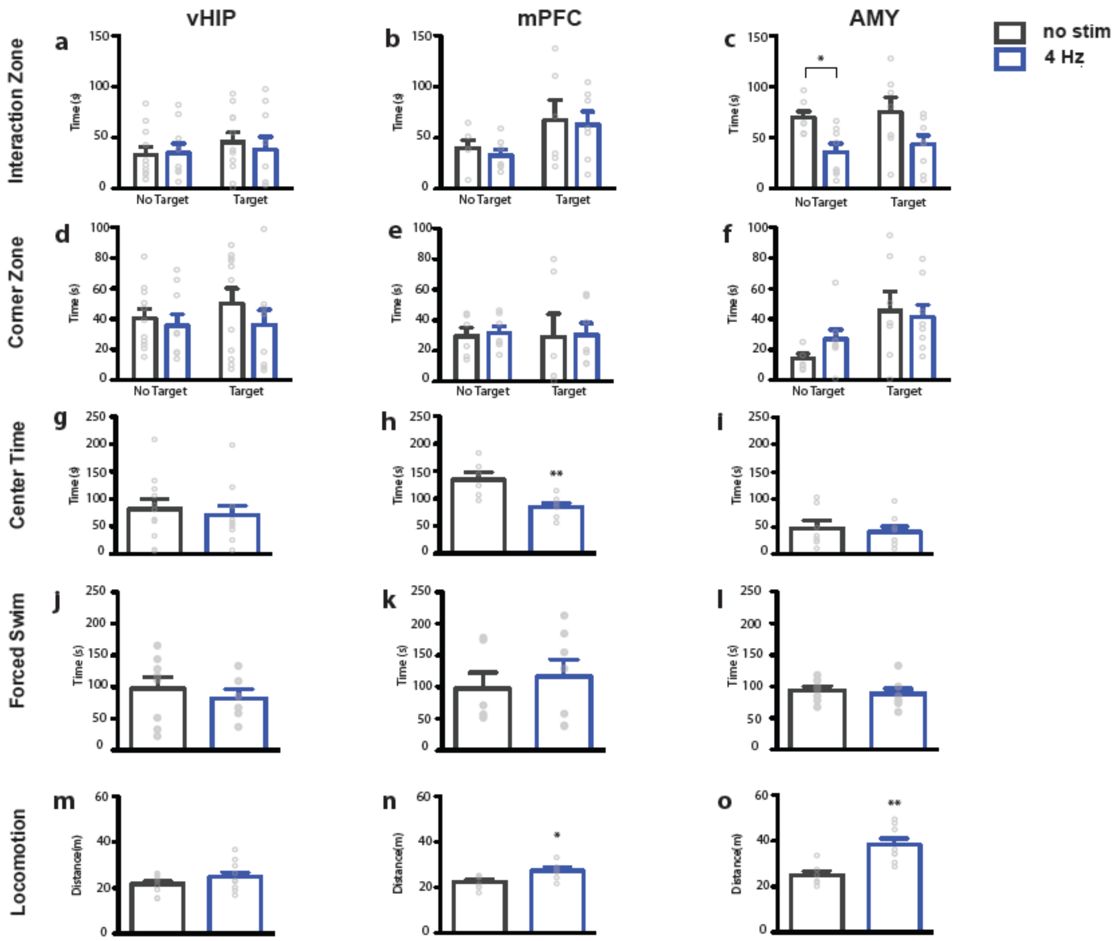

<u>Supplementary Figure 6.</u> Acute stimulation of vHIP, mPFC or AMY terminals in NAc does not affect social interaction in stress-naïve mice. In non-defeated stress-naïve mice, 4 Hz stimulation of terminals in NAc shell arising from vHIP (a; n=11,8) or mPFC (b; n=6,7) did not alter time in interaction zone or time in corners (d-f). 4 Hz stimulation of AMY (c) terminals in NAc non-specifically increased time spent in the interaction zone ($F_{1,13}$=7.291 main effect of stimulation, p<0.05, *post-hoc* *p<0.05, n=7,8). vHIP (g) terminal stimulation did not affect anxiety-like behavior in the OFT (n=11,10). (h) Stimulation of mPFC terminals increased anxiety behavior, reflected by decreased time spent in the center of an OFT (t=3.39, *p<0.05). AMY (f) axon stimulation did not alter anxiety behavior in the OFT. Afferent stimulation did not alter immobility in the forced swim test (j-l). Unlike vHIP (m; n=11,10), stimulation of (n) mPFC (t=2.64, *p<0.05) or (o) AMY (t=3.87, **p<0.01) terminals increased distance traveled in the OFT. (a-f) Analyses by two-way repeated measures ANOVAs. P-values refer to analyses by student's t-tests for two group comparisons. 'No stim' and '4 Hz' denote non-stimulated control mice and 4 Hz stimulated mice, respectively.

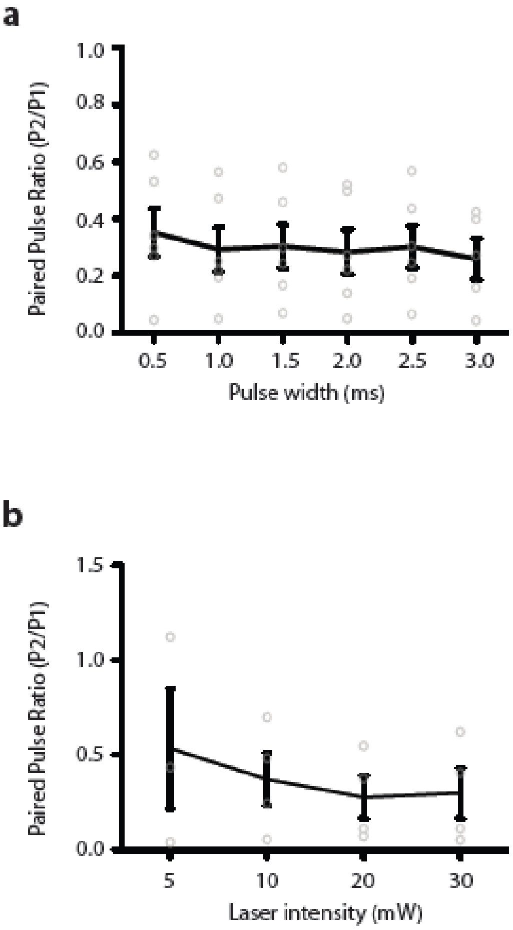

<u>Supplementary Figure 7.</u> Stability of paired pulse ratio across pulse width and laser intensity. Paired pulse ratio (P_2/P_1) (100 ms interpulse interval) of optically evoked EPSCs in NAc shell MSNs was stable across a range of (a) pulse widths (n=7) and (b) laser intensities (n=4) although increased variability in paired pulse ratio was observed at low laser intensities (5 mW) far below the intensity level used in the present work (30 mW).
